# The role of *Staphylococcus aureus* lipoproteins in hematogenous septic arthritis

**DOI:** 10.1038/s41598-020-64879-4

**Published:** 2020-05-13

**Authors:** Majd Mohammad, Zhicheng Hu, Abukar Ali, Pradeep Kumar Kopparapu, Manli Na, Anders Jarneborn, Mariana do Nascimento Stroparo, Minh-Thu Nguyen, Anna Karlsson, Friedrich Götz, Rille Pullerits, Tao Jin

**Affiliations:** 1 Department of Rheumatology and Inflammation Research, Institute of Medicine, Sahlgrenska Academy, University of Gothenburg, Gothenburg, Sweden; 2 Department of Microbiology and Immunology, The Affiliated Hospital of Guizhou Medical University, Guiyang, China; 3 Department of Rheumatology, Sahlgrenska University Hospital, Gothenburg, Sweden; 4 Department of Microbial Genetics, University of Tübingen, Tübingen, Germany; 5 Department of Clinical Immunology and Transfusion Medicine, Sahlgrenska University Hospital, Gothenburg, Sweden

**Keywords:** Bacterial host response, Bacterial infection

## Abstract

Permanent joint dysfunction is a devastating complication in patients with septic arthritis. *Staphylococcus aureus* (*S. aureus*) lipoproteins (Lpp), the predominant ligands for TLR2, are known to be arthritogenic and induce bone destruction when introduced directly into the joint. Here, we aim to investigate the importance of *S. aureus* Lpp and TLR2 in a hematogenous septic arthritis model, which is the most common route of infection in humans. C57BL/6 wild-type and TLR2 deficient mice were intravenously inoculated with *S. aureus* Newman parental strain or its lipoprotein-deficient *Δlgt* mutant strain. The clinical course of septic arthritis, radiological changes, and serum levels of cytokines and chemokines, were assessed. Newman strain induced more severe and frequent clinical septic polyarthritis compared to its *Δlgt* mutant in TLR2 deficient mice, but not in wild-type controls. Bone destruction, however, did not differ between groups. Lpp expression was associated with higher mortality, weight loss as well as impaired bacterial clearance in mouse kidneys independent of TLR2. Furthermore, Lpp expression induced increased systemic pro-inflammatory cytokine and neutrophil chemokine release. Staphylococcal Lpp are potent virulence factors in *S. aureus* systemic infection independent of host TLR2 signalling. However, they have a limited impact on bone erosion in hematogenous staphylococcal septic arthritis.

## Introduction

Septic arthritis remains a devastating and invasive joint disease. Due to its rapidly progressing nature, septic arthritis is considered a medical emergency^1^ with a poor prognosis. Despite advances in understanding and treatment of infectious diseases, the prospect of patients with septic arthritis has remained poor. Almost half of the patients will suffer from permanent joint destruction^2^, if treatment is not initiated immediately^3^. The estimated incidence of septic arthritis in the general population is approximately 6–10 cases per 100,000 individuals per year^4^. However, in patients with an underlying joint disease, such as rheumatoid arthritis (RA), the incidence of septic arthritis is nearly 10 times higher than in the general population^4^.

Septic arthritis is most often caused by *Staphylococcus aureus* (*S. aureus*), a pathogenic Gram-positive bacterium^5^. *S. aureus*-induced septic arthritis has been extensively studied for the past few decades; several virulence factors as well as various host-factors targeted by the bacterium have been identified^6–11^. However, much still remains elusive regarding the bacteria-host interaction in *S. aureus* septic arthritis.

Staphylococcal lipoproteins (Lpp), important bacterial molecules in *S. aureus*, consist of a lipid-moiety and a protein-part, and are anchored in the bacterial cytoplasmic membrane^12^. Lpp are important for bacterial survival during infection due to their role in maintaining the metabolic activity of the bacteria^13,14^. The lipid structure of Lpp is known to stimulate the innate immune system through activation of pattern recognition receptors^15^, and bacterial Lpp are predominant ligands for Toll-like receptor 2 (TLR2)^16–18^. The role of staphylococcal Lpp in *S. aureus* infections has been studied in different infection models, including sepsis and skin infection^6,19–22^.

In a recent study, we demonstrated that purified *S. aureus* Lpp, when injected intra-articularly into the mouse knee joints, induced destructive arthritis in TLR2-dependent manner^6^. However, it is well-known that the majority of septic arthritis in patients is caused by hematogenous spreading of bacteria^11^. It still remains unclear whether staphylococcal Lpp enhance disease severity in hematogenous septic arthritis. In the present study, we investigated the role of staphylococcal Lpp as well as TLR2 in our well-established hematogenous mouse model of *S. aureus*-induced septic arthritis. Our findings demonstrate that expression of staphylococcal Lpp increases the virulence of *S. aureus* systemic infection, independently of TLR2, but their effect on radiological bone erosion is limited.

## Results

### Staphylococcal lipoproteins induce more severe and frequent clinical septic polyarthritis in TLR2 deficient mice

**Figure 1:**
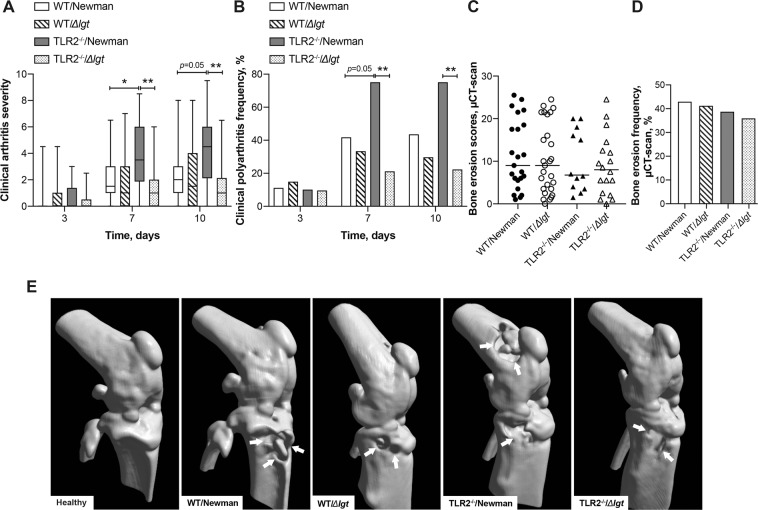
Staphylococcal lipoproteins induce more severe and frequent septic polyarthritis in TLR2 deficient mice. C57BL/6 wild-type mice (WT) were intravenously inoculated with a dose of 4.0–6.0 × 10^6^ colony forming units of *S. aureus* Newman parental strain (WT/Newman; n = 27) or Newman*Δlgt* mutant strain (WT/*Δlgt*; n = 27), and Toll-like receptor 2 deficient (TLR2^−/−^) mice were intravenously inoculated with the same dose of *S. aureus* Newman parental strain (TLR2^−/−^/Newman; n = 21) or Newman*Δlgt* mutant strain (TLR2^−/−^/*Δlgt*; n = 21). The clinical severity of arthritis (**A**), the frequency of polyarthritis (**B**), the bone erosion scores (**C**), and cumulative frequency of bone erosion (**D**) evaluated with a microcomputed tomography (μCT) scan on day 10 post-infection. Representative μCT scan images (**E**) of mouse knee joints (healthy C57BL/6 wild-type mouse; WT/Newman; WT/*Δlgt*; TLR2^−/−^/Newman; and TLR2^−/−^/*Δlgt*, on day 10 post-infection. Arrows indicate bone erosion. The data were pooled from 3 independent experiments. Statistical evaluations were performed using the Mann–Whitney U test, with data expressed as box plots showing medians and interquartile range, and whiskers showing minimum and maximum (A), the median (C), or Fisher’s exact test (B and D). *P < 0.05; **P < 0.01.

To study the importance of *S. aureus* Lpp and the influence of TLR2 deficiency on the severity and frequency of clinical arthritis, wild-type (WT) and TLR2 deficient (TLR2^−/−^) mice were intravenously inoculated with an arthritic dose of either *S. aureus* Newman parental strain expressing Lpp (WT/Newman and TLR2^−/−^/Newman, respectively) or *S. aureus* Newman*Δlgt* mutant strain (WT/*Δlgt* and TLR2^−/−^/*Δlgt*, respectively). With regards to the effect of Lpp, we observed that the Newman parental strain induced significantly more severe clinical arthritis than the *Δlgt* mutant strain in the TLR2^−/−^ mice on days 7 (*P* = 0.002) and 10 (*P* = 0.002) post-infection (Fig. 1A). However, no tangible differences with respect to arthritis severity were observed between the bacterial strains within the WT mouse groups during the course of infection (Fig. 1A). Interestingly, with regards to the effect of TLR2, we observed that TLR2^−/−^ mice inoculated with the Newman parental strain developed significantly more severe clinical arthritis than the WT mice infected with the same strain (*P* = 0.02 on day 7, and *P* = 0.05 on day 10; Fig. 1A), whereas there was no difference when *Δlgt* mutant strain was used, indicating that TLR2 deficiency aggravates arthritis only in the presence of Lpp. Furthermore, the same trends were observed in the mice with regards to the frequency of clinical polyarthritis. Newman parental strain caused a higher incidence of polyarthritis in TLR2^−/−^ mice than the *Δlgt* mutant strain on day 7 (75% vs 21.1%; *P* = 0.002) and on day 10 (75% vs 22.2%; *P* = 0.008). In addition, TLR2^−/−^ mice exhibited a tendency towards higher incidence of polyarthritis compared to the WT mice on day 7 post-infection when they were infected with the Newman parental strain (75% vs 41.7%; *P* = 0.05; Fig. 1B).

To detect bone destruction of septic arthritis, a microcomputed tomography (µCT)-scan was utilized to examine the mouse joints. The µCT revealed that all the infected groups displayed similar severity of accumulative bone destruction score on the termination day (Fig. 1C). In line with these results, 43% of the joints from WT/Newman group developed radiological bone destructions compared to 41% in the WT/*Δlgt* group, whereas 39% developed radiological bone destructions in the TLR2^−/−^/Newman group compared to 36% in the TLR2^−/−^/*Δlgt* group (Fig. 1D,E). Interestingly, a more detailed subgroup analysis of bone destruction revealed that the hind paws in the WT/*Δlgt* group exhibited a tendency of more severe bone destruction than the TLR2^−/−^/*Δlgt* group (*P* = 0.05; see Supplementary Table S1). However, no other differences were observed between the remaining subgroups of joints in terms of severity or frequency of bone destruction (see Supplementary Table S1 and Supplementary Table S2).

### Deficiency in prelipoprotein lipidation in *S*. *aureus* decreases mortality in mice with staphylococcal septic arthritis

**Figure 2:**
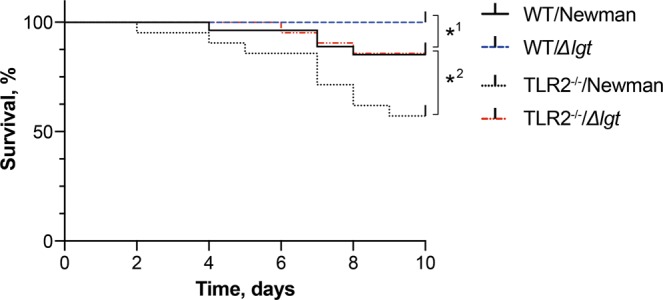
Deficiency in prelipoprotein lipidation in *S*. *aureus* decreases mortality in mice with staphylococcal septic arthritis. Log rank survival curve showing survival rate comparison between C57BL/6 wild-type mice (WT) intravenously inoculated with a dose of 4.0–6.0 × 10^6^ colony forming units of *S. aureus* Newman parental strain (WT/Newman; n = 27) or Newman*Δlgt* mutant strain (WT/*Δlgt*; n = 27), and Toll-like receptor 2 deficient (TLR2^−/−^) mice intravenously inoculated with the same dose of *S. aureus* Newman parental strain (TLR2^−/−^/Newman; n = 21) or Newman*Δlgt* mutant strain (TLR2^−/−^/*Δlgt*; n = 21). The surviving mice were euthanized on day 10 post-infection. The data were pooled from 3 independent experiments. Statistical evaluations were performed using the log-rank (Mantel-cox) test. ^1^ = WT/Newman vs. WT/*Δlgt*; ^2^ = TLR2^−/−^/Newman vs. TLR2^−/−^/*Δlgt*; ^*^P < 0.05.

The arthritogenic dose of *S. aureus* Newman parental strain and *Δlgt* mutant strain were used to further assess the lethality among the groups during the course of 10 days. The overall survival rate was lower in mice infected with Newman parental strain as compared to *Δlgt* mutant strain (Fig. 2). The survival rate among the WT/Newman group was 85%, whereas 100% of mice in the WT/*Δlgt* group survived (*P* = 0.04) to the end of the experiment. Newman parental strain also increased the mortality in TLR2^−/−^ mice. The TLR2^−/−^ mice infected with Newman parental strain had the highest mortality among all of the groups with a survival rate of only 57%, compared to its *Δlgt* counterpart which had a survival rate of 86% (*P* = 0.04). On the whole, the TLR2^−/−^ mice had significantly higher mortality compared to their WT counterparts, regardless of the bacterial strain (*P* < 0.05; Fig. 2).

These results indicate that expression of staphylococcal Lpp induce higher mortality, while TLR2 seems to have a protective role in *S. aureus*-induced septic arthritis.

### Staphylococcal lipoproteins are associated with increased weight loss in mice with staphylococcal septic arthritis

**Figure 3:**
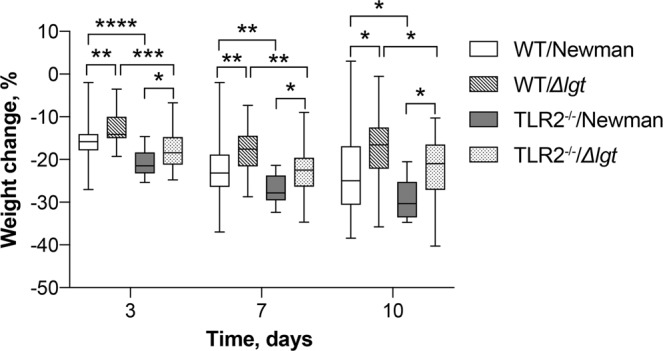
Staphylococcal lipoproteins are associated with increased weight loss in mice. Changes in percentage of body weight in C57BL/6 wild-type mice (WT) intravenously inoculated with a dose of 4.0–6.0 × 10^6^ colony forming units of *S. aureus* Newman parental strain (WT/Newman; n = 27) or Newman*Δlgt* mutant strain (WT/*Δlgt*; n = 27), and Toll-like receptor 2 deficient (TLR2^−/−^) mice intravenously inoculated with the same dose of *S. aureus* Newman parental strain (TLR2^−/−^/Newman; n = 21) or Newman*Δlgt* mutant strain (TLR2^−/−^/*Δlgt*; n = 21), and were monitored up to 10 days post-infection. The data were pooled from 3 independent experiments. The box plots show medians and interquartile range, whiskers show minimum and maximum. Statistical evaluations were performed using the Mann–Whitney U test. *P < 0.05; **P < 0.01; ***P < 0.001; ****P < 0.0001.

After inoculation, mice infected with the Newman parental strain lost significantly more weight than mice infected with the Newman*Δlgt* mutant strain throughout the course of the experiment (Fig. 3). Intriguingly, this pattern was observed among both the WT mice as well as the TLR2^−/−^ mice infected with the Newman parental strain compared to their counterparts infected with the *Δlgt* mutant strain on all monitored time points (*P* < 0.05; Fig. 3). In line with the increased mortality in TLR2^−/−^ mice, the weight reduction was significantly more pronounced among the TLR2^−/−^ mice in comparison to the WT mice infected with the corresponding bacterial strain (Fig. 3).

### Staphylococcal lipoproteins and TLR2 deficiency impair bacterial clearance in mice

Bacterial persistence in kidneys reflects the capacity of the host immune system to eliminate bacteria, and is thus an important parameter in our animal model.

**Figure 4:**
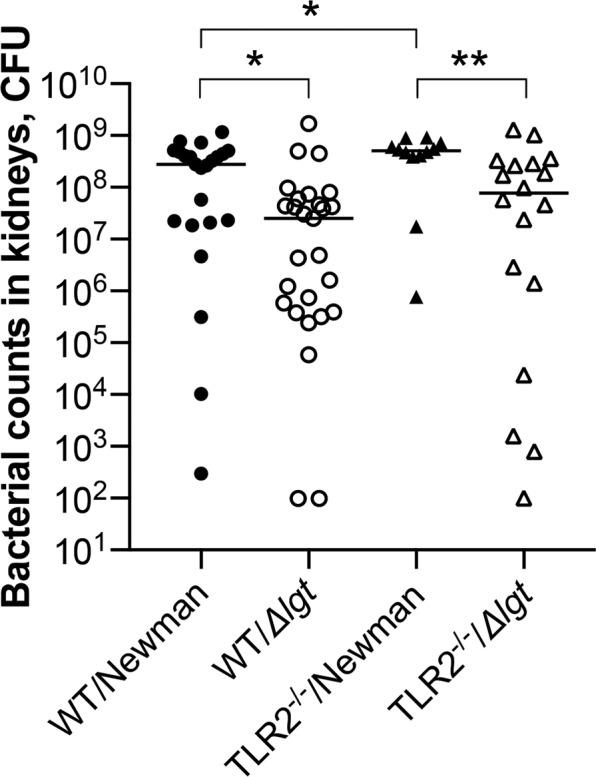
Staphylococcal lipoproteins and TLR2 deficiency impair bacterial clearance in mice. Persistence of *S. aureus* in kidneys of C57BL/6 wild-type mice (WT) intravenously inoculated with a dose of 4.0–6.0 × 10^6^ colony forming units of *S. aureus* Newman parental strain (WT/Newman; n = 23) or Newman*Δlgt* mutant strain (WT/*Δlgt*; n = 27), and Toll-like receptor 2 deficient (TLR2^−/−^) mice intravenously inoculated with the same dose of *S. aureus* Newman parental strain (TLR2^−/−^/Newman; n = 12) or Newman*Δlgt* mutant strain (TLR2^−/−^/*Δlgt*; n = 18) on day 10 post-infection. The data were pooled from 3 independent experiments. Statistical evaluations were performed using the Mann–Whitney U test, with data expressed on a logarithmic scale as the median. *P < 0.05; **P < 0.01.

*S. aureus* Newman parental strain increased the bacterial burden, in comparison to the Newman*Δlgt* strain, among both the WT (*P* = 0.01) and the TLR2^−/−^ mice groups (*P* = 0.008) on day 10 post-infection (Fig. 4), suggesting that staphylococcal Lpp enhances the bacterial survival in mice, which is independent of TLR2. Moreover, TLR2^−/−^ mice infected with Newman parental strain displayed significantly worse bacterial clearance than WT mice inoculated with the same strain (*P* = 0.04; Fig. 4), thus indicating that TLR2 deficiency impairs the host’s ability to clear the bacteria.

### *S. aureus* lipoproteins induce increased pro-inflammatory cytokine and neutrophil chemokine release

**Figure 5:**
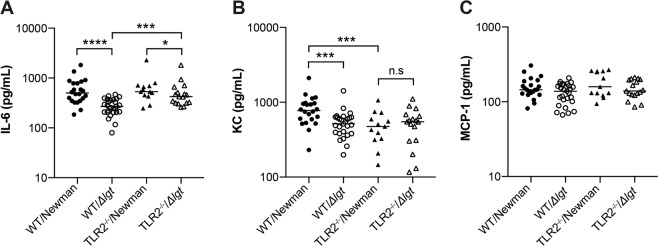
*S. aureus* lipoproteins induce increased pro-inflammatory cytokine and neutrophil chemokine release. The levels of (**A**) Interleukin-6 (IL-6), (**B**) Keratinocyte chemoattractant (KC), and (**C**) Monocyte chemoattractant protein 1 (MCP-1) in the sera collected from C57BL/6 wild-type mice (WT) intravenously inoculated with a dose of 4.0–6.0 × 10^6^ colony forming units of *S. aureus* Newman parental strain (WT/Newman; n = 22) or Newman*Δlgt* mutant strain (WT/*Δlgt*; n = 27), and Toll-like receptor 2 deficient (TLR2^−/−^) mice intravenously inoculated with the same dose of *S. aureus* Newman parental strain (TLR2^−/−^/Newman; n = 12) or Newman*Δlgt* mutant strain (TLR2^−/−^/*Δlgt*; n = 18) on day 10 post-infection. The data were pooled from 3 independent experiments. Statistical evaluations were performed using the Mann–Whitney U test, with data expressed on a logarithmic scale as the median. *P < 0.05; ***P < 0.001; ****P < 0.0001; n.s = not significant.

To elucidate the systemic inflammatory response in mice induced by staphylococcal infection, serum levels of various cytokines and chemokines were analysed (Fig. 5A–C). Both WT and TLR2^−/−^ mice inoculated with the Newman parental strain had significantly higher levels of the pro-inflammatory cytokine IL-6 (*P* < 0.0001 and *P* < 0.05, respectively) than those infected with Newman*Δlgt*. TLR2^−/−^ mice had significantly higher IL-6 levels than WT mice only when *Δlgt* mutant strain was used (*P* < 0.001). Strikingly, WT mice inoculated with the Newman parental strain had higher levels of a neutrophil attracting chemokine KC (*P* < 0.001) than those infected with Newman*Δlgt*, whereas no difference was observed when TLR2^−/−^ mice were used, suggesting that *S. aureus* Lpp induce KC release through TLR2. No difference was found among the groups regarding the serum levels of monocyte-attracting chemokine MCP-1.

### The impact of lipoprotein deficiency on bacterial growth and competition in different conditions

**Figure 6:**
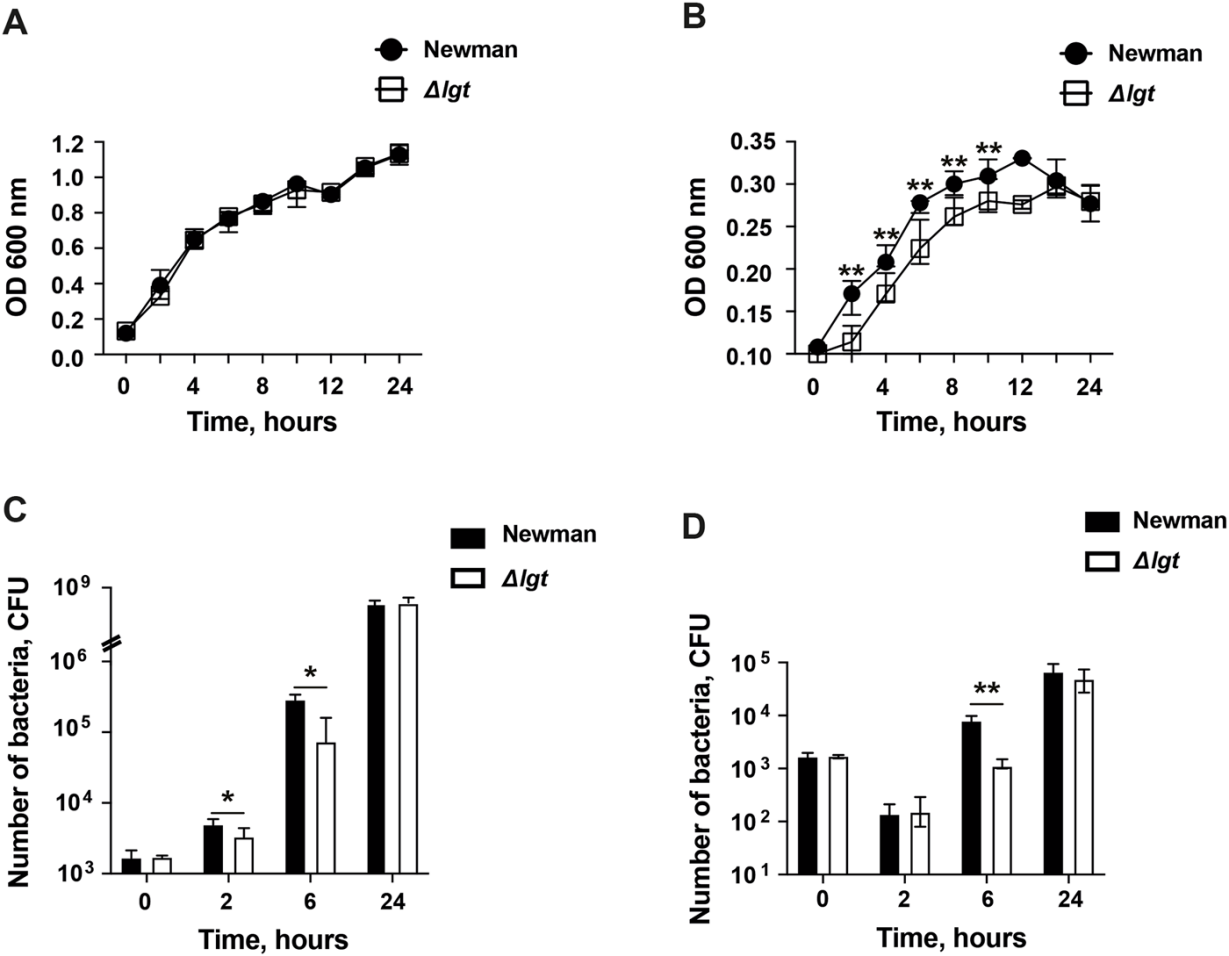
Impact of lipoprotein deficiency on bacterial growth and competition in different conditions. Growth of *S. aureus* Newman parental strain or Newman*Δlgt* mutant strain alone after each bacterial solution were adjusted to a starting concentration of 0.1 using optical density at 600 nm (OD 600 nm) in (**A**) tryptic soy broth (TSB) medium and (**B**) RPMI-1640 medium. The data were pooled with duplicate samples from each group from 3 independent experiments. Mixtures of both Newman parental strain and Newman*Δlgt* mutant strain with an initial concentration of 1500 colony forming units (CFU)/ml of each strain in (**C**) TSB medium and (**D**) RPMI-1640 medium. At specific time intervals, samples of the bacterial mixtures were evaluated by comparing the number of CFUs on horse blood agar plates and trypticase soy agar (TSA) plates with erythromycin (2.5 µg/ml). The data were pooled from 5 independent experiments. Statistical evaluations were performed using the Mann–Whitney U test, with data expressed as the median with 95% CI. *P < 0.05; **P < 0.01.

To compare the growth rate between the staphylococcal Newman parental strain and its *Δlgt* mutant strain, the strains were cultured separately in tryptic soy broth (TSB) and RPMI medium. Both strains exhibited an equal growth pattern throughout the 24-hour incubation in the nutrient rich TSB medium (Fig. 6A). However, in the RPMI medium, which is nutrient poor, the growth was significantly more pronounced during the first 10 hours of incubation in the Newman parental strain (*P* < 0.01; Fig. 6B), suggesting that Lpp expression is more advantageous for the bacteria in the early phase of bacterial growth in the nutrient poor condition.

Next, we assessed whether the Newman parental strain has the ability to outcompete the *Δlgt* mutant strain by performing a competition assay. To do so, both strains were titrated to the same concentration of 1500 colony forming units (CFU)/ml, mixed and cultured in both TSB as well as RPMI medium and the CFU counts were followed for up to 24 hours of incubation. Interestingly, the parental strain outcompeted the *Δlgt* mutant strain at 2- and 6 hours of incubation in TSB medium (*P* < 0.05; Fig. 6C), but only at 6 hours of incubation in the RPMI medium (*P* < 0.01; Fig. 6D). The growth rate became similar at 24 hours of bacterial culture in both TSB and RPMI medium.

### Impact of lipoprotein deficiency on expression of virulence factors

**Figure 7:**
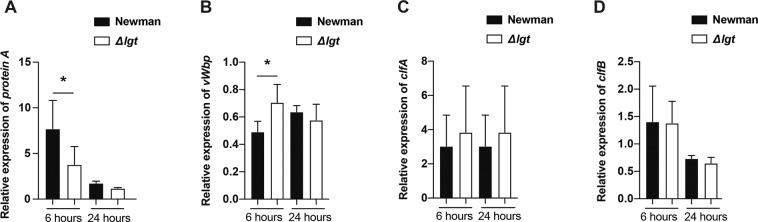
*S. aureus* lipoprotein expression displays similar transcription levels as its *lgt*-deficient mutant strain. Transcriptional levels of *S. aureus* genes through a culture period of 6- or 24 hours in tryptic soy broth (TSB) medium with Newman parental strain or Newman*Δlgt* mutant strain after extraction of total RNA: (**A**) protein A, (**B**) von Willebrand factor-binding protein (*vWbp*), (**C**) clumping factor A (*clfA*), and (**D**) clumping factor B (*clfB*). The relative expression levels of each gene to gyrase B (*gyr*B) were compared by quantitative Real-Time PCR. The data were pooled from the average of the triplicates of 4 independent experiments. Statistical evaluations were performed using the two-tailed Student’s t-test, with data expressed as the mean ± standard error of the mean. *P < 0.05.

In order to address whether the expression levels of various *S. aureus* virulence factors are influenced due to staphylococcal Lpp deficiency, total RNA from both strains were isolated at two different time points after culturing the bacteria in TSB medium (Fig. 7A–D). Intriguingly, the Newman parental strain exhibited higher expression level of protein A than the Newman*Δlgt* mutant strain from the 6-hour bacterial culture (*P* < 0.05). In contrast, the parental strain displayed lower expression level of von Willebrand factor-binding protein (*vWbp*) than its mutant strain at the same time point (*P* < 0.05). No tangible differences were observed between the bacterial strains with regards to clumping factor A (*clfA*) or clumping factor B (*clfB*).

## Discussion

In this study, we investigated the effect of staphylococcal Lpp and TLR2 in a murine model of *S. aureus*-induced hematogenous septic arthritis. Our results demonstrate that Lpp expression has no impact on clinical septic arthritis in wild-type mice. However, in TLR2 deficient mice, Lpp is a strong virulence factor, giving rise to more severe arthritis. In contrast to significantly aggravated clinical arthritis, no difference was found with regards to bone erosion. In addition, staphylococcal Lpp play a potent role in weight loss, overall mortality, and bacterial clearance in mice, which is independent of host TLR2 signalling. Importantly, the combination of Lpp expression (Newman parental strain) and lack of TLR2 lead to the most severe disease outcome, underlining the importance of TLR2 for protection in the hematogenous arthritis model.

Previously, we showed that staphylococcal Lpp are one of the major arthritogenic bacterial components causing destructive arthritis in wild-type mice but not in TLR2 deficient mice in an animal arthritis model of intra-articular injection of bacterial components^6^. In the current study, a more clinically relevant animal model (hematogenous septic arthritis model) was applied. Our results suggest that Lpp have limited impact on the development of hematogenous septic arthritis in WT mice, whereas TLR2 deficiency gives more severe clinical septic arthritis, which is somehow contradictory to our previous findings. There are several important aspects in terms of the implemented experimental settings between these two studies that need to be highlighted: 1) intact live *S. aureus* bacteria vs. purified staphylococcal Lpp; and 2) systemic staphylococcal infection model vs. local knee joint model.

**Figure 8:**
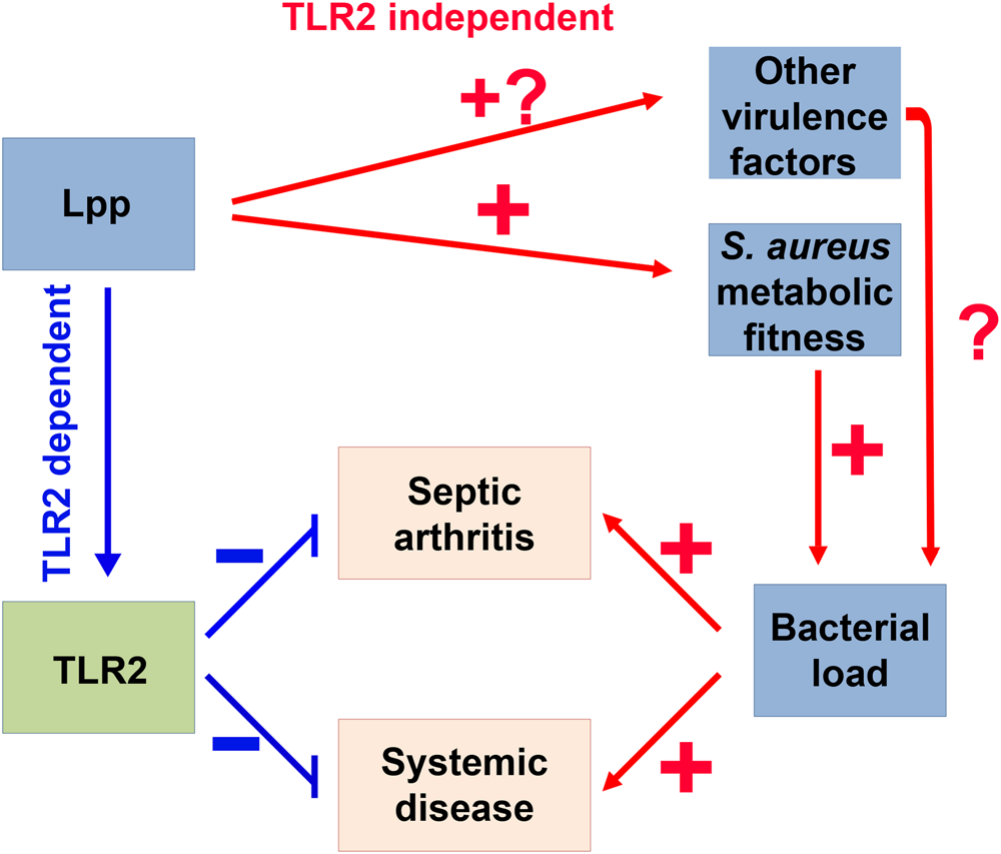
Schematic illustration of possible mechanism of *S. aureus* lipoproteins in hematogenous septic arthritis. *S. aureus* lipoproteins (Lpp) play the dual roles in hematogenous septic arthritis. On one hand, staphylococcal Lpp arouse a protective immune response through TLR2. On the other hand, they maintain an improved metabolic fitness compared to the *Δlgt* mutant strain, consequently leading to higher bacterial load in the organs and more severe disease, which is TLR2 independent. The clinical outcome of an infection is an overall result of both ‘good’ and ‘bad’ effects induced by Lpp.

Our results clearly demonstrate that it makes a difference when you apply Lpp directly into joints that causes a TLR2-dependent bone erosion^6^ – or – when you intravenously infect mice with living bacterial cells, Lpp-producing or non-producing. When infecting mice with living bacteria, many other factors come into play, such as the fitness and survival in the host, or innate and adaptive immune activation or evasion^14,15^. Hereby, we propose two different mechanisms for the clinical outcomes observed in our current study – TLR2 dependent and TLR2 independent (Fig. 8). On one hand, staphylococcal Lpp arouse a protective immune response through TLR2. Indeed, various *S. aureus*-induced murine infection models demonstrate that lack of TLR2 leads to a more severe disease outcome, resulting in higher persistence of bacterial burdens in organs^20,23–26^. On the other hand, it is well-known that staphylococcal Lpp are crucial for iron acquisition, independent of host TLR2 expression, which is essential for better survival in infections^20^. Due to the dual roles of Lpp, the clinical outcome of an infection is therefore an overall result of both ‘good’ and ‘bad’ effects induced by Lpp. In case of *S. aureus* parental strain infection in TLR2 deficient mice, there were only ‘bad’ effects – increased metabolic fitness that enhanced the bacterial load but without TLR2-mediated protection, which resulted in the most severe outcome. In the case of *Δlgt* mutant infection in WT mice, no increased metabolic fitness and normal TLR2 mediated immune responses (two ‘good’ effects) led to the milder disease. For the other two combinations, both ‘good’ and ‘bad’ effects exist and encounter each other. Therefore, the overall disease outcome became moderate. Also, we speculate that the metabolic fitness mechanism might be more predominant compared to TLR2 dependent mechanism in our model system, since in both wild-type and TLR2 deficient mice, Newman parental strain displayed better survival in kidneys than *Δlgt* mutant. Interestingly, we have also observed that the expression of certain virulence factors in the *lgt*-deficient mutant strain differ from the parental strain. Protein A was downregulated whereas vWbp was upregulated in the early hours of culture. The clinical relevance of the altered gene expression is unclear and the deeper mechanism needs to be investigated in the future studies.

Unexpectedly, the clinical arthritis severity did not correlate to the radiological findings in our current study. There are several possible explanations for this discrepancy. Firstly, we observed the high mortality rate in TLR2 deficient mice infected with the Newman parental strain. Of note, most of the mice that succumbed to the disease in the TLR2^−/−^/Newman group exhibited severe clinical arthritis, which were radiologically not possible to examine due to the deaths of animals prior to the study endpoint. Secondly, TLR2 deficient mice, lacking staphylococcal Lpp ligand recognition, are more sensitive to *S. aureus* infection, which lead to more severe and frequent clinical septic polyarthritis. However, since Lpp is predominant bone erosion inducer in septic arthritis and the bone erosive effect of Lpp is strictly dependent on TLR2^6^, bone destruction tended to be less pronounced in the TLR2 deficient mice compared to wild-type mice despite displaying more clinical septic arthritis. In other inflammatory conditions, articular bone erosion due to excess generation of osteoclast-mediated local bone resorption and inadequate bone formation is mainly induced by increased expression of cytokines and receptor activator of nuclear factor κB ligand (RANKL)^27^. In fact, a synthetic lipopeptide, mimicking the structure of bacterial Lpp has previously been shown to stimulate osteoclast formation *in vivo* by a TLR2-dependent mechanism^28^. Moreover, the importance of lipopeptides in inducing local and systemic bone loss in mice has also been reported^29^.

The host innate immunity rapidly recognizes intruding pathogens and serves as a first line of defence to the host through activation of polymorphonuclear neutrophils and mononuclear phagocytes together with initiation of a quick release of inflammatory cytokines and chemokines^30^. Mice depleted of neutrophils displayed significantly higher mortality and more severe arthritis, demonstrating the protective role of neutrophils in host response to invading *S. aureus* in murine septic arthritis^31^. In contrast to neutrophils, monocytes/macrophages play a vital role in the pathogenesis of *S. aureus* septic arthritis by aggravating the severity of arthritis in mice^32^. Indeed, the progression of septic arthritis was strongly mediated by monocytes/macrophages through TLR2, but not by neutrophils, in staphylococcal Lpp-induced knee joint arthritis^6^. Chemokines, such as neutrophil chemoattractants KC and MIP-2, are crucial recruiters of neutrophils^33^. Previous studies demonstrated the rapid release of KC in murine peritoneal macrophages upon stimulation by purified staphylococcal Lpp through TLR2^6^. This is in line with our current hematogenous staphylococcal septic arthritis study; Lpp triggered the systemic release of KC to a significantly higher extent than those of the *lgt*-deficient mutant strain, possibly mediated via TLR2. The release of monocyte chemokine MCP-1, on the other hand, was not dependent on Lpp induction.

In summary, we conclude that *S. aureus* Lpp are potent virulence factors in *S. aureus* systemic infection regardless of host TLR2 signalling. However, Lpp have a limited impact on radiological bone erosion in hematogenous staphylococcal septic arthritis.

## Methods

### Ethics statement

Mouse studies were reviewed and approved by the Ethics Committee of Animal Research of Gothenburg. Mouse experiments were conducted in accordance with recommendations listed in the Swedish Board of Agriculture’s regulations and recommendations on animal experiments.

### Mice

C57BL/6 wild-type mice and Toll-like receptor 2-deficient B6.129-Tlr2^tm1Kir^/J (TLR2^−/−^) mice were purchased from Charles River Laboratories (Sulzfeld, Germany) and The Jackson Laboratory (Bar Harbor, Maine, USA), respectively. All mice were bred and housed in the animal facility of the Department of Rheumatology and Inflammation Research, University of Gothenburg. Mice were kept under standard temperature and light conditions and were fed laboratory chow and water *ad libitum*. The Ethics Committee of Animal Research of Gothenburg approved the study, and the animal experimentation guidelines were strictly followed.

### Bacterial strains and preparation of bacterial solutions

*S. aureus* Newman strain, and its lipoprotein-deficient *Δlgt* mutant strain, Newman*Δlgt* mutant, generated exactly as previously described^19^, were cultured separately on horse blood agar plates or trypticase soy agar (TSA) plates with erythromycin (2.5 µg/ml) for 24 hours, respectively. Afterwards, the bacteria were harvested, and stored at −20 °C in phosphate-buffered saline (PBS) containing 5% bovine serum albumin (BSA) and 10% dimethyl sulfoxide (DMSO). Before each experiment, the bacterial solutions were thawed, washed in PBS, and adjusted to the required concentration.

### Bacterial growth conditions

The growth kinetics of *S. aureus* Newman parental strain and Newman*Δlgt* mutant strain were compared in both nutrient rich- and nutrient poor conditions using TSB and RPMI-1640 medium (Fisher Scientific, Waltham, MA, USA), respectively. Initially, Newman parental strain as well as Newman*Δlgt* mutant strain were grown overnight in horse blood agar plate or TSA plate with erythromycin (2.5 µg/ml), respectively. Afterwards, a single colony of the respective bacterial strain was inoculated in both TSB and RPMI-1640 medium. The bacterial solutions were adjusted to a concentration of 0.1 using optical density at 600 nm (OD_600_), and thereafter incubated at 37 °C. At different time intervals, duplicate samples were collected to assess the concentration. The results from three independent experiments displayed a similar pattern and the data were pooled.

### Bacterial growth competition assay

For the growth competition assay, 1500 CFU/ml of both *S. aureus* Newman parental strain and Newman*Δlgt* mutant strain were mixed in both TSB and RPMI medium and incubated at 37 °C. At specific time intervals, the samples of the bacterial mixtures (100 μl) were serially diluted in PBS and spread on horse blood agar plates as well as TSA plates with erythromycin (2.5 µg/ml).

To determine the number of colonies from the respective strain, the difference in CFU counts between the two different plates were used since only the Newman*Δlgt* mutant strain grows in both types of plates. To ensure that the Newman parental strain do not grow on TSA plates with erythromycin, the bacteria was plated and incubated for 24 hours. No bacterial growth was detected. The results from five independent experiments displayed a similar pattern and the data were pooled.

### Gene expression of virulence factors

Newman parental strain and Newman*Δlgt* mutant strain were grown overnight in horse blood agar plate or TSA plate with erythromycin (2.5 µg/ml), respectively. Afterwards, a single colony of the respective bacterial strain was inoculated in TSB medium and incubated at 37 °C. After 6 and 24 hours, bacterial solution from each strain were withdrawn, and centrifuged at 4000 rpm for 10 min at 4 °C. The resulting pellet was washed in PBS and 1 ml of QIAzol Lysis Reagent (217004; Qiagen, Hilden, Germany) was added. To ensure efficient bacterial cell disruption and total RNA release, glass beads (G4649; Sigma-Aldrich, Saint Louis, MO, USA) were added into each tube and processed with a frequency of 30/s for 10 min in TissueLyser II (Qiagen, Hilden, Germany).

Total RNA was extracted using miRNeasy Mini Kit (217004; Qiagen, Hilden, Germany) according to the manufacturer’s protocol. Reverse transcription was performed using the iScript cDNA Synthesis Kit (1708890; Bio-Rad, Hercules, CA, USA) according to the manufacturer’s instructions using Thermal Cycler Veriti instrument (Applied Biosystems, Foster City, CA, USA) with the following settings: 5 min at 25 °C, 20 min at 46 °C, 1 min at 95 °C, followed by addition of 1 μl (2 units) of RNase H (part of Superscript III first-strand synthesis supermix for quantitative Real-Time PCR; 11752050; Invitrogen, Carlsbad, CA, USA). Quantitative Real-time PCR was performed using the ViiA 7 system instrument (Applied Biosystems, Foster City, CA, USA) using TaqMan gene expression assay for *protein A* (1787866 C10; Cat No: 4332079; Applied Biosystems, Foster City, CA, USA); *clfA* (1787866 C8; Cat No: 4332079; Applied Biosystems, Foster City, CA, USA); *clfB* (1787866 C9; Cat No: 4332079; Applied Biosystems, Foster City, CA, USA); and *vWbp* (1787866 C11; Cat No; 4332079, Applied Biosystems, Foster City, CA, USA) according to manufacturer’s instructions. Gyrase B (*gyr*B) (1789527 C5; Cat No: 4332079; Applied Biosystems, Foster City, CA, USA) was used as an internal control. All samples were run in triplicates. The relative expression of each gene to *gyr*B (the standard reference control) was calculated using the ΔCt method. The results were pooled from the average of the triplicates of four independent experiments.

### Experimental protocol for staphylococcal septic arthritis

To study the impact of both staphylococcal lipoproteins as well as TLR2 on staphylococcal septic arthritis, three separate *in vivo* experiments were performed using wild-type (n = 14–24/experiment) and TLR2^−/−^ mice (n = 10–18/experiment). Half of the wild-type and TLR2^−/−^ mice were infected with the Newman parental strain, while the other half were infected with the Newman*Δlgt* mutant.

In all experiments, mice were intravenously inoculated with 0.2 ml of the respective bacterial strain suspensions into the tail vein with an arthritogenic dose of 4.0–6.0 × 10^6^ CFUs per mouse. The mice were monitored individually: survival was assessed daily; mice were regularly weighed and examined for the presence of clinical arthritis. The animals were sacrificed if deemed not to survive until the next scheduled time point and considered dead. All mice were euthanized on day 10 post-infection, and serum samples, kidneys, and the paws were collected and stored as previously described^9^. The results from those three independent experiments displayed a similar pattern and the data were pooled.

### Clinical evaluation of arthritis

Observers (M.M. and Z.H.), blinded to the genetic background of the mice, visually evaluated all 4 limbs of each mouse on days 3, 7, and 10 post-infection. Arthritis was defined as erythema and/or swelling of the joints. A clinical scoring system ranging from 0 to 3 points was used for each paw, as described before^9,34^. The arthritis index was constructed by adding the scores from all 4 limbs of each mouse, whereby each animal could be awarded a maximum of 12. The clinical polyarthritis was defined as arthritis in ≥2 of the paws of an individual mouse.

### Measurement of cytokine and chemokine levels

The levels of IL-6, MCP-1, and KC in sera were quantified using DuoSet ELISA kits (R&D Systems, Abingdon, UK) according to the instructions from manufacturers.

### Microcomputed tomography (µCT)

Imaging and processing of all limb joints of the mice to detect bone destruction after the studies were terminated were performed with a Skyscan1176 µCT scanner (Bruker, Antwerp, Belgium), as previously described^34^. The 3-dimensional images were reconstructed using NRECON software (version 1.6.9.8; Bruker) and analysed with CT-Analyzer (version 2.7.0; Bruker). Afterwards, the extent of bone and cartilage destruction was assessed in a blinded manner by experienced observers (M.M. and Z.H.) using a grading scale from 0–3 as previously described^34^. Severity index of bone erosions was calculated by adding the scores of all 6 scanned joint areas from left and right side of the mouse (forepaws and wrists, elbows, shoulders, back paws and ankles, knees, and hips), whereby each animal could be awarded a maximum score of 36 points. Joints with a score of ≥1 were considered positive in terms of bone erosion frequency.

### Statistical analysis

Statistical significance was assessed using the Mann-Whitney U test, Fisher’s exact test, and log-rank (Mantel-cox) test, as appropriate. Differences in gene expression between groups were assessed using the two-tailed Student’s t-test. A *P* value <0.05 was considered statistically significant. Calculations were performed using GraphPad Prism version 8.0.2 software for Macintosh (GraphPad software, La Jolla, CA, USA).

## Supplementary information

Supplementary information.

## Data Availability

The datasets generated during and/or analysed during the current study are available from the corresponding author on reasonable request.
