# Supplementary material for: The role of Staphylococcus aureus lipoproteins in hematogenous septic arthritis
Source: Sci Rep. 2020 May 13;10:7936. doi: 10.1038/s41598-020-64879-4 (PMC7221087; doi:10.1038/s41598-020-64879-4)
Supplement: Supplementary file 1 — Supplementary information. [file 41598_2020_64879_MOESM1_ESM.pdf]

Supplementary Information

The role of *Staphylococcus aureus* lipoproteins in hematogenous septic arthritis

Majd Mohammad[1], Zhicheng Hu[1,2], Abukar Ali[1], Manli Na[1], Anders Jarneborn[1,3], Mariana do Nascimento Stroparo[1], Pradeep Kumar Kopparapu[1], Minh-Thu Nguyen[4], Anna Karlsson[1], Friedrich Götz[4], Rille Pullerits[1,5], Tao Jin[1,3]

1. Department of Rheumatology and Inflammation Research, Institute of Medicine, Sahlgrenska Academy, University of Gothenburg, Gothenburg, Sweden

2. Department of Microbiology and Immunology, The Affiliated Hospital of Guizhou Medical University, Guiyang, China

3. Department of Rheumatology, Sahlgrenska University Hospital, Gothenburg, Sweden

4. Department of Microbial Genetics, University of Tübingen, Tübingen, Germany

5. Department of Clinical Immunology and Transfusion Medicine, Sahlgrenska University Hospital, Gothenburg, Sweden

Supplementary Table S1. Subgroup analysis of bone destructions by a micro-computed tomography scan.

	Severity (Mean ± SEM)			
	WT/Newman	WT/*Δlgt*	TLR2$^{-/-}$/Newman	TLR2$^{-/-}$/*Δlgt*
Hind paws	0.40±0.10	0.45±0.09 †	0.50±0.14	0.19±0.07
Knees	1.84±0.18	1.73±0.16	1.98±0.27	1.78±0.19
Hips	0.86±0.17	0.74±0.15	0.58±0.21	0.57±0.17
Front paws	0.64±0.15	0.66±0.13	0.46±0.20	0.44±0.12
Elbows	0.21±0.9	0.05±0.03	0.00±0.00	0.10±0.06
Shoulders	1.74±0.17	1.64±0.17	1.48±0.24	1.39±0.22

The data are reported as mean ± SEM and analyzed with the Mann-Whitney U test. † p=0.05 versus TLR2$^{-/-}$/*Δlgt*. WT/Newman= C57BL/6 wild-type mice infected with Newman wild-type strain; WT/*Δlgt*= C57BL/6 wild-type mice infected with Newman*Δlgt* mutant strain; TLR2$^{-/-}$/Newman= TLR2 deficient mice infected with Newman wild-type strain; TLR2$^{-/-}$/*Δlgt*= TLR2 deficient mice infected with Newman*Δlgt* mutant strain.

Supplementary Table S2. Subgroup analysis of bone destructions by a micro-computed tomography scan.

	Frequency (%)			
	WT/Newman	WT/*Δlgt*	TLR2$^{-/-}$/Newman	TLR2$^{-/-}$/*Δlgt*
Hind paws	32.6	37.0	37.5	19.4
Knees	80.4	77.8	79.2	80.6
Hips	39.1	35.2	29.2	27.8
Front paws	30.4	38.9	20.8	30.6
Elbows	13.0	3.7	0.0	8.3
Shoulders	76.1	72.2	75.0	58.3

Statistical evaluations were performed using the Fisher's exact test. WT/Newman= C57BL/6 wild-type mice infected with Newman wild-type strain; WT/*Δlgt*= C57BL/6 wild-type mice infected with Newman*Δlgt* mutant strain; TLR2$^{-/-}$/Newman= TLR2 deficient mice infected with Newman wild-type strain; TLR2$^{-/-}$/*Δlgt*= TLR2 deficient mice infected with Newman*Δlgt* mutant strain.
